# Supplementary material for: Molecular Characterization of HOXA2 and HOXA3 Binding Properties
Source: J Dev Biol. 2021 Dec 3;9(4):55. doi: 10.3390/jdb9040055 (PMC8707757; doi:10.3390/jdb9040055)
Supplement: Supplementary file 1 [file jdb-09-00055-s001.zip › jdb-1447626-supplementary.pdf]

Supplementary Material

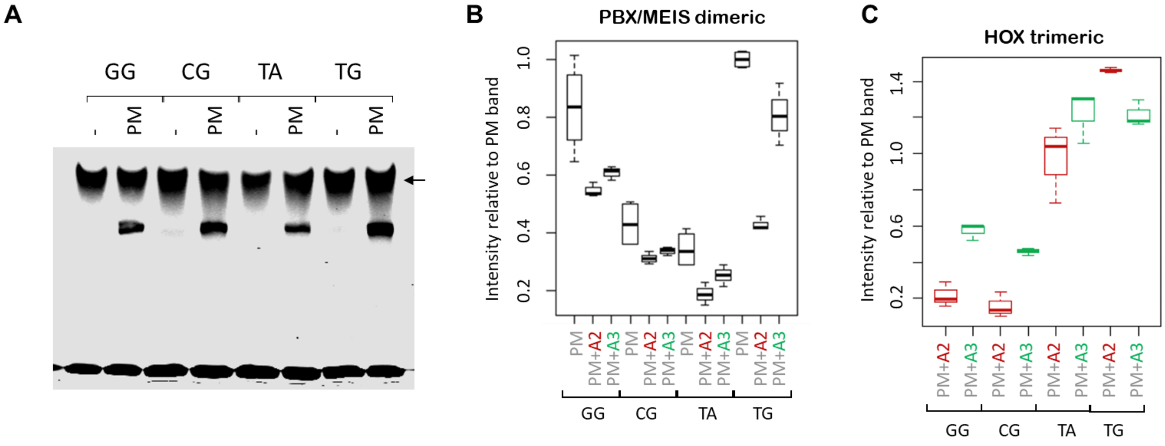

Figure S1. A. Binding to the *Meis2* probes detailed in Fig. 1B, in the presence and absence of PBX/MEIS (PM). All probes tested displayed the same nonspecific binding (black arrow). B. Quantification of PBX/MEIS (PM) band from experiments in Fig 1C and 2B (3 replicates). PBX/MEIS band in the absence of HOXA reduced in CG and TA mutants relative to wild-type. All bands quantified relative to the wild-type PBX/MEIS band. C. Quantification of HOXA/PBX/MEIS bands from experiments in Fig 1C and 2B (3 replicates). In contrast to wild-type, CG and GG mutants have stronger binding of HOXA3 with PBX/MEIS than HOXA2.

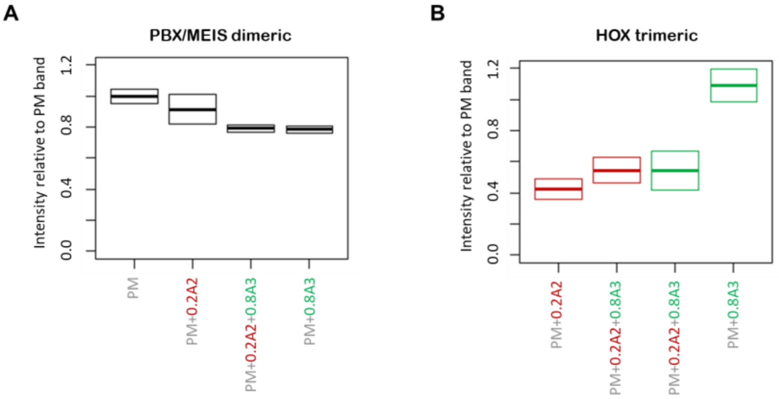

Figure S2. A. Quantification of PBX/MEIS (PM) band from experiments in Fig 4B (2 replicates). B. Quantification of HOXA/PBX/MEIS bands from experiments in Fig 4B (2 replicates). HOXA3 binding is reduced in the presence of HOXA2, but a 4x excess of HOXA3 is unable to displace HOXA2.

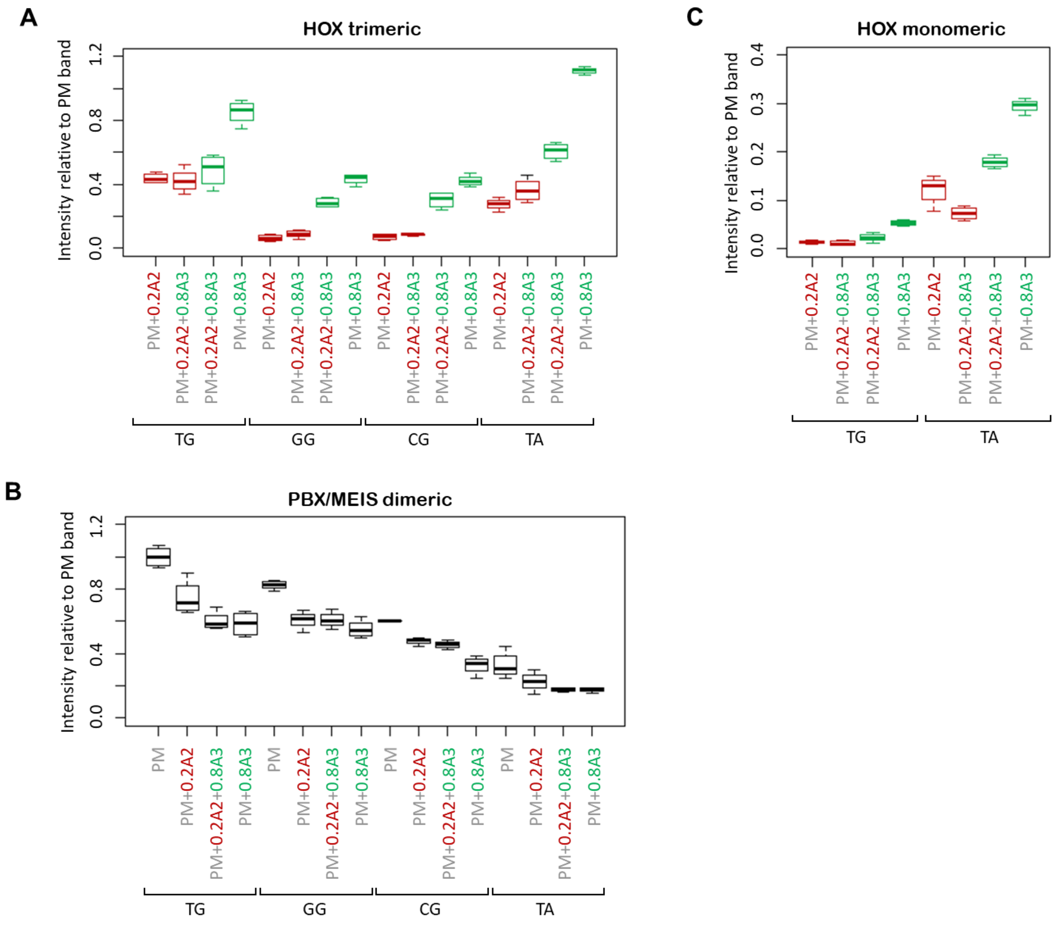

Figure S3. A. Quantification of HOXA/PBX/MEIS bands from experiment in Fig 4C (3 replicates). B. Quantification of PBX/MEIS (PM) band from experiment in Fig 4C (3 replicates). C. Quantification of HOXA bands from experiment in Fig 4C (3 replicates).
